# Paternal Uniparental Disomy of the Entire Chromosome 20 in a Child with Beckwith-Wiedemann Syndrome

**DOI:** 10.3390/genes12020172

**Published:** 2021-01-27

**Authors:** Sanaa Choufani, Jung Min Ko, Youliang Lou, Cheryl Shuman, Leona Fishman, Rosanna Weksberg

**Affiliations:** 1 Program in Genetics and Genome Biology, The Hospital for Sick Children, Toronto, ON M5G 0A4, Canada; sanaa.choufani@sickkids.ca (S.C.); jmkogenetics@gmail.com (J.M.K.); Youliang.lou@sickkids.ca (Y.L.); 2 Department of Pediatrics, Seoul National University Children’s Hospital, Seoul National University College of Medicine, Seoul 03080, Korea; 3 Department of Molecular Genetics, University of Toronto, Toronto, ON M5G 1X8, Canada; Cheryl.shuman@utoronto.ca; 4 Division of Clinical and Metabolic Genetics, The Hospital for Sick Children, Toronto, ON M5G 1X8, Canada; leona.fishman@sickkids.ca; 5 Department of Pediatrics, University of Toronto, Toronto, ON M5S 1A1, Canada

**Keywords:** Beckwith-Wiedemann syndrome, pseudohypoparathyroidism type 1b, genomic imprinting, uniparental disomy, DNA methylation, *GNAS*

## Abstract

Epigenetic alterations at imprinted genes on different chromosomes have been linked to several imprinting disorders (IDs) such as Beckwith-Wiedemann syndrome (BWS) and pseudohypoparathyroidism type 1b (PHP1b). Here, we present a male patient with these two distinct IDs caused by two independent mechanisms-loss of methylation (LOM) at chromosome 11p15.5 associated with multi-locus imprinting disturbances (MLID and paternal uniparental disomy of chromosome 20 (patUPD20). A clinical diagnosis of BWS was made based on the clinical features of macrosomia, macroglossia, and umbilical hernia. The diagnosis of PHP1b was supported by the presence of reduced growth velocity and mild learning disability as well as hypocalcemia and hyperphosphatemia at 14 years of age. Molecular analyses, including genome-wide DNA methylation (Illumina 450k array), bisulfite pyrosequencing, single nucleotide polymorphism (SNP) array and microsatellite analysis, demonstrated loss of methylation (LOM) at IC2 on chromosome 11p15.5, and paternal isodisomy of the entire chromosome 20. In addition, imprinting disturbances were noted at the differentially methylated regions (DMRs) associated with *DIRAS3* on chromosome 1 and *PLAGL1* on chromosome 6. This is the first case report of PHP1b due to patUPD20 diagnosed in a BWS patient with LOM at IC2 demonstrating etiologic heterogeneity for multiple imprinting disorders in a single individual.

## 1. Introduction

Beckwith–Wiedemann syndrome (BWS, OMIM#130650) and pseudohypoparathyroidism (PHP, OMIM#103580, #603233, #612463) are genetic imprinting disorders (IDs), caused by genetic and epigenetic alterations involving imprinting control regions (ICs) and differentially methylated regions (DMRs) on chromosomes 11p15.5 and 20q13.32, respectively. BWS is the most common genetic overgrowth syndrome involving a predisposition to embryonal tumor development in childhood. The clinical presentation in BWS is variable and findings may include neonatal hypoglycemia, macrosomia, macroglossia, abdominal wall defects, visceromegaly, renal abnormalities, cytomegaly of the fetal adrenal cortex, ear creases and/or pits, embryonal tumor, etc [1,2]. PHP includes a number of conditions associated primarily with target organ tissue resistance to parathyroid hormone (PTH) leading to hypocalcemia and hyperphosphatemia. Its pathogenesis has been linked to dysfunctional Gs α subunit of G proteins and PHP can be divided into several clinical subgroups by clinical, molecular, and biochemical characteristics (PHP1A, PHP1B, PHP1C and PHP2) [3,4,5]. Clinical features associated with PHP disorders are variable and may include skeletal findings, small stature, and intellectual disability among others [5].

The molecular defects resulting in both BWS and PHP are heterogeneous. Alterations of imprinting processes in two ICs, IC1 and IC2, on chromosome 11p15 can be identified in approximately 70–80% of clinically diagnosed BWS patients. In approximately 50% of the cases, the molecular defect consists in loss of methylation of IC2 (IC2-LOM) on the maternal copy of chromosome 11 [1,2,6]. PHP is associated with pathogenic variants and/ or methylation defects within the imprinted *GNAS* cluster on 20q13.32 are associated with PHP [7]. Among the subtypes of PHP, PHP1b (OMIM#603233) is defined as end-organ resistance to multiple hormones including PTH and the mostly absence of features of Albright’s hereditary osteodystrophy (AHO) [3]. The consistent defect in PHP1b is paternal-specific patterns within all four DMRs on *GNAS* cluster of maternal inherited alleles showing simultaneous gain of methylation (GOM) at *NESP55* DMR and LOM at *AS, XL*, and *A/B* DMRs [8]. The molecular mechanism remains largely undefined in PHP1b, and complete or segmental uniparental disomy of paternally derived chromosome 20 (patUPD20) is identified as the cause of this broad imprinting defect only in a small subset of sporadic PHP1b patients [9,10,11,12,13].

Moreover, it has been recently revealed that molecular disturbances are not restricted to the disease specific region, but affect other chromosomal loci in some patients with IDs including BWS and PHP1b [14,15,16]. This phenomenon is referred to as multilocus imprinting disturbance (MLID). MLID has been identified in BWS patients at frequencies of up to 30% in patients with LOM at IC2 [17], and the *GNAS* cluster is one of the commonly affected genes for MLID in BWS patients with IC2-LOM [18]. Additional methylation alterations on *GNAS* leading to clinical phenotypes of PHP1B besides those of BWS have been reported in two female patients [16,19]. However, the underlying cause for MLID also remains to be determined, although a few pathogenic variants in genes such as *ZFP57,* important for DNA methylation maintenance post-fertilization, and in specific maternal effect genes such as *NLRP2,* and *PADI6* have been reported in a few ID patients with MLID [20,21,22,23].

Here, we report a new male patient with clinical phenotypes of two distinct IDs, sporadic BWS with IC2-LOM and sporadic PHP1b due to patUPD20 involving the entire chromosome 20, as well as MLID.

## 2. Materials and Methods

### 2.1. DNA Methylation Analysis Using the Illumina 450k Array

The research was approved by the Research Ethics board at The Hospital for Sick Children and consent was obtained from participating individuals and /or their parents or guardians. The study was conducted in accordance with the Declaration of Helsinki, and the protocol was approved by the Ethics Committee (REB# 1000038847). Peripheral blood genomic DNA from six control samples and the patient DNA sample were sodium bisulfite converted using the Qiagen EZ DNA Methylation kit (Qiagen, Valencia, CA), according to the manufacturer’s protocol. Modified genomic DNA was then processed and analyzed on the Infinium HumanMethylation450 BeadChip from Illumina (Illumina 450K, San Diego, CA, USA). The data was analyzed for differential methylation at imprinted loci as previously described [24].

#### Normalization and Quality Controls

We used the GenomeStudio software from Illumina to process the raw intensity data (IDAT files) for all samples. Control normalization and background subtraction included in GenomeStudio were used to generate DNA methylation profiles, or β values, for each sample at every CpG site from the ~485,000 CpG sites. All samples passed the quality controls measures and had over 485,000 CpG sites detected at a detection *p*-value < 0.01. Further quality controls were used to exclude probes overlapping chromosomes X and Y, probes containing single nucleotide polymorphisms (SNPs with MAF > 1% that is within 5bp of single base extension site, and low signal probes (if more than a third of samples with Detection *p*-value > 0.01) as previously described [24].

Epigenetic alterations at imprinted genes have previously been associated with BWS. Therefore, we focused our analysis on 648 CpG sites that overlap known differentially methylated regions of imprinted loci in the genome identified on the 450k array [24]. DNA methylation profiles of the proband were compared against six control samples run in the same microarray batch. Differential DNA methylation analysis at each individual DMR was performed by comparing the mean methylation values for each DMR to the mean of controls plus or minus 2SD as previously described [24].

### 2.2. Chromosomal Microarray with Affymetrix 6.0 SNP Array

Genomic DNA from the patient and his parents were processed on the Affymetrix Genome-Wide Human SNP Array 6.0 at The Center for Applied Genomics (TCAG, Toronto) as previously described [25]. Copy number variations (CNVs) and loss of homozygosity at chromosome 20 were performed using the Genotyping Console software from Affymetrix.

### 2.3. Polymorphic Microsatellite Analysis for Chromosome 20

Analysis of genomic DNA from the patient and his parents was performed using polymorphic microsatellite markers throughout the chromosome 20 to confirm UPD20. We used 16 microsatellite markers distributed throughout the entire chromosome 20 (for chromosome 20p: D20S117, D20S889, D20S115, D20S186, D20S875, D20S885; for chromosome 20q: D20S195, D20S884, D20S107, D20S96, D20S119, D20S838, D20S178, D20S840, D20S100, D20S171). A fluorescently labeled forward primer and a conventional reverse primer were used for PCR, and PCR products were run on an ABI3130xl Genetic analyzer (Applied Biosystems, Foster city, CA, USA). Alleles were analyzed using GenMarker version 1.7 (SoftGenetics, State College, PA, USA) at the Center for Applied Genomics (Toronto).

## 3. Results

### 3.1. Clinical Presentation

The patient is the second child of nonconsanguineous Caucasian parents. He was conceived naturally and delivered vaginally at 40 weeks gestation. The birth weight was 4.26 kg (+1.40 SD, 92th percentile). The pregnancy was uncomplicated, and prenatal ultrasonography was normal. At delivery, both parents were 39 years old, and the elder sister was three years old. There was no family history of genetic syndromes, congenital anomalies, or thyroid disorders. For first five days after birth, the proband was monitored in the neonatal intensive care unit. He had cyanosis associated with shoulder dystocia during delivery, as well as hypoglycemia, macroglossia, and a large umbilical hernia. Intravenous glucose was used to stabilize his blood glucose. Macroglossia interfered with breast feeding and he was switched to bottle feeds during hospitalization. At the age of 6 weeks, elevated TSH and low normal free T4 levels were noted, and levothyroxine therapy was started. At the age of three months, he was evaluated in the Genetics Clinic for macrosomia and macroglossia. His height, weight, and head circumference were 63.5 cm (+2.5 SD, >97th percentile), 7.2 kg (+3.5 SD, >97th percentile), and 43.5 cm (+3.1 SD, >97th percentile), respectively. He was noted to have a coarse facial appearance, macroglossia, a large umbilical hernia, and a crease on the left earlobe. No additional features associated with BWS were noted on examination. Abdominal ultrasound was normal. Conventional G-banding karyotype of peripheral leukocytes was normal (46, XY), and did not indicate any gross structural change of chromosomes. Clinical MS-MLPA testing was done on peripheral blood and identified IC2-LOM, without DNA copy number changes on chromosome 11p15. A clinical diagnosis of BWS was established and he underwent recommended tumor surveillance via his pediatrician, i.e., three monthly abdominal ultrasound to the age of eight years and AFP measurement to the age of four years. This surveillance did not identify any abnormal findings throughout the recommended time frame. At the age of 2.5 years, he underwent tongue reduction surgery for macroglossia. His thyroid function was monitored and was maintained within the normal range with levothyroxine and liothyroxine replacement. A mild learning disability was diagnosed at the age of six years and an individualized education program was provided to him.

**Figure 1:**
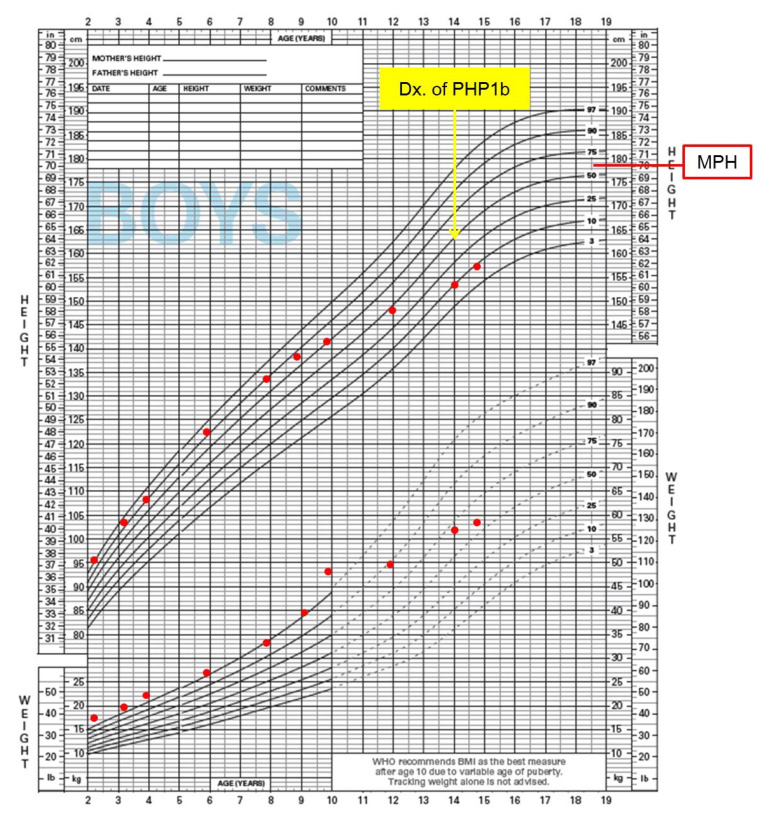
Patient growth curve. In his growth curve, the height velocity had been maintained above 90th percentile until about 8 years old, however the height velocity had slowed down after 9–10 years old and locates below the range of genetic target height (MPH) at 14 years of age.

At the age of 14 years, the proband was again seen in the Genetics Clinic (Figure 1). He did not have a history of hypocalcemia or tetany. His height and weight were 153.7 cm (−1.3 SD, 10th percentile) and 56.7 kg (+0.5 SD, 70th percentile), respectively and he had normal body proportions without evidence of skeletal abnormalities. His midparental height was 178.6 cm (+0.2 SD, 60th percentile). His external genitalia were Tanner stage II, Neurologic examination was normal except for brisk deep tendon reflexes on his knees. His thyroid function was within normal ranges, while blood chemistry analysis revealed hypocalcemia and elevated PTH levels. Levels of serum magnesium, alkaline phosphatase, and 25-hydroxyvitamin D were normal. In addition to BWS, he was diagnosed with PHP1b was diagnosed. He was immediately treated with intravenous calcium replacement followed by oral calcium and vitamin D therapy. There was no evidence of nephrocalcinosis on abdominal ultrasound. He has since completed his high school education and is attending college at this time.

### 3.2. Identification of MLID Using the 450k Aray

DNA methylation alterations at several imprinted genes are known to be associated with BWS. Therefore, we extracted from the Illumina 450k array, 648 CpG sites that overlap known differentially methylated regions of imprinted loci in the genome (Supplementary Table S1). We compared the DNA methylation profiles of the proband at these imprinted CpG sites against six control samples run in the same microarray batch. Differential DNA methylation analysis at each individual DMR was performed by comparing the mean methylation values for each DMR to the mean of controls plus or minus 2SD as previously described [24]. We identified CpG methylation alterations at multiple imprinted loci in the proband including *DIRAS3* (1p31.3), *PLAGL1* (6q24), *KCNQ10T1* (IC2, 11p15.5), and the *GNAS* (20q13.32) cluster (Table 1). That is, in addition to the IC2-LOM associated with the primary diagnosis of BWS, the proband demonstrated MLID. In the GNAS cluster on chromosome 20, all maternally derived imprinted DMRs showed loss of methylation. Interestingly, in the *GNAS* cluster, the only paternally methylated DMR, *NESP55* DMR, showed gain of methylation in contrast to loss of methylation at the other maternally methylated DMRs within the GNAS cluster (*XL* and *A/B* DMRs). This configuration of methylation alterations across the *GNAS* cluster is the commonly observed pattern in sporadic PHP1b including patUPD20. No methylation alterations were observed at other paternally methylated loci i.e., *H19, MEG3,* and *ZNF597* DMRs (Supplementary Table S1).

### 3.3. Identification of Loss-of-Heterozygosity and Paternal Isodisomy of Chromosome 20

**Figure 2:**
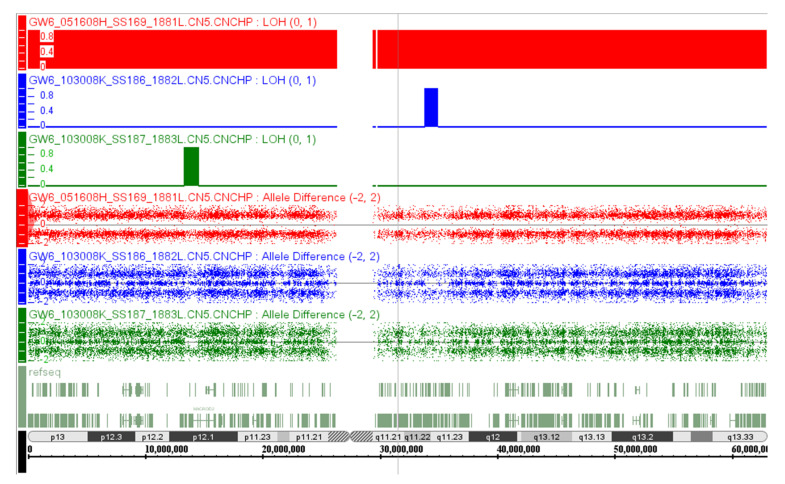
SNP array findings for chromosomes. Analysis of genomic DNA from the patient and his parents was performed using Affymetrix 6.0 array and identified loss of heterozygosity in the proband (**red**) compared to his mother (**green**) and the father (**blue**). Regions of LOH greater than 3 Mb are summarized in the red block. Note that the LOH in the proband extends across the entire chromosome 20. These data further validated the presence of uniparental disomy of the entire chromosome 20 in the BWS proband.

**Figure 3:**
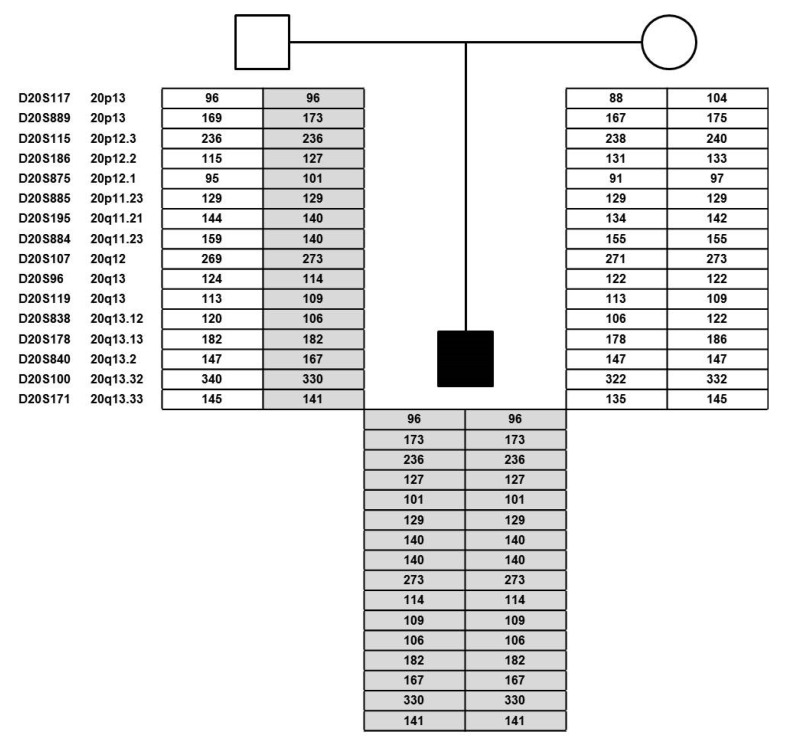
Polymorphic microsatellite marker analysis. Pedigree of the proband and his parents showing the results of the 16 polymorphic microsatellite markers distributed throughout the entire chromosome 20 (6 markers for chromosome 20p and 10 markers for chromosome 20q). This data confirms that the patient harbors isodisomy of the entire chromosome 20 originated from his father. Grey areas indicate the region of UPD and its parental origin.

To determine whether the abnormal DNA methylation pattern observed at the chromosome 20 imprinting DMRs is caused by uniparental disomy (UPD), we performed copy number analysis on the proband and his parents using the Affymetrix 6.0 SNP array. Microarray analysis revealed a maternally inherited deletion on chr2:228,272,835-228,318,628 (hg18) with a deletion size of 45,794 bp. This deletion overlaps with the promoter region and part of the coding region of the SLC19A3 gene. Mutations of bilateral SLC19A3 alleles lead to thiamine metabolism dysfunction syndrome-2 (OMIM#607483) with progressive encephalopathy mainly affecting the basal ganglia. No change was found in copy number for chromosome 20. However, homozygosity screening showed copy neutral loss of heterozygosity (LOH) affecting the entirety of chromosome 20 (Figure 2). These findings indicated that the two chromosomes 20 are identical and the patient has UPD for chromosome 20. These findings were also validated using polymorphic microsatellite markers distributed across chromosome 20. The result shows loss of heterozygosity (LOH) with lack of maternal contribution at all informative markers (Figure 3). These data validated the initial DNA methylation findings supporting the presence of paternal uniparental isodisomy for chromosome 20 associated with PHP1b as well as IC2-LOM on chromosome 11p15.5 and MLID associated with BWS.

## 4. Discussion

We report here the first male patient with two clinically distinct imprinting disorders, BWS and PHP-1b, caused by two independent molecular mechanisms. The features of BWS were recognizable in infancy, while PHP-1b was diagnosed in adolescence. The molecular alterations identified in this patient confirmed his clinical diagnoses.

Using MS-MLPA, 11p15 LOM at IC2 was identified, a molecular change associated with ~50% of BWS cases. The proband showed additional methylation defects at *DIRAS3* and *PLAGL1* along with those involving IC2 and the *GNAS* cluster. We also demonstrated that the PHP1b was associated with methylation alterations at the GNAS cluster and was caused by pat UPD 20 rather than MLID; whereas the other methylation alterations at IC2, *DIRAS3* and *PLAGL1* were not associated with UPD and were attributable to MLID.

To our knowledge, this is the first case of a patient with BWS associated with LOM at IC2 and PHP1b with patUPD20. Although 80% of PHP1b is sporadic and most sporadic cases show a broad range of methylation alterations at the *GNAS* cluster, only a small subset (~8%) of sporadic PHP1b is due to patUPD20 [26]. Since the first description of patUPD20q as a cause of a sporadic case of PHP1b [9], less than 15 patients with PHP1b due to patUPD20 have been reported [11,12,13,27,28,29]. Most of these patients showed isodisomy of the long arm of paternal chromosome 20, and only one patient revealed isodisomy of the entire chromosome 20 originating from the father [27]. Our patient is the second reported case of paternal isodisomy of the entire chromosome 20. It is most likely that BWS with MLID and PHP1b due to patUPD20 are the result of independent molecular events rather than a common unknown underlying mechanism.

MLID associated with the IC2 BWS molecular subtype can demonstrate various types of generalized imprinting alterations [14,15,16] and 25–34% of BWS patients with IC2 LOM demonstrate MLID [30,31]. In PHP1b, 6–12% of patients showed MLID at several imprinted loci other than *GNAS* [30,32]. The *GNAS* cluster is one of the frequent sites affected by MLID in BWS patients, whereas PHP1b patients rarely have methylation alterations at IC1 or IC2 on chromosome 11p15 as part of MLID [14,32,33].

To data, MLID has always been associated with LOM at more than one imprinted DMRs whereas reports of gain of methylation (GOM) at imprinted DMRs are rare. The latter findings are almost never associated with UPD [14,15,16]. Moreover, there has been no report of MLID observed in PHP1b patients with patUPD20. Most BWS or PHP1b patients with MLID are indistinguishable from those without MLID in terms of clinical features including growth parameters and biochemical values [15,31,32]. However, epigenotype-phenotype correlation in patients with MLID revealed a significant sex bias, with a male-to-female ratio of 1:4 [18]. Some patients have been reported to manifest clinical features of two different IDs in the presence of MLID. Specifically, there are two female patients reported with both BWS and PHP1b phenotypes as part of their MLID molecular findings (Table 2) [19,22]. Both of these female patients had IC2-LOM for BWS as well as diffuse imprinting defects which mimic the paternal-specific methylation pattern (LOMs of *AS, XL, A/B* DMRs, and GOM of *NESP55* DMR) on *GNAS* cluster for PHP1b. Importantly, copy number alterations as well as UPD were excluded by SNP array, microsatellite analysis, and/or MS-MLPA, and in case 2 (Table 2) genetic mutations in genes previously associated with MLID were also excluded [19,22]. Notably, the proband reported here is the first male patient reported with IC2-LOM for BWS and a paternal UPD20 as well as clinical features of BWS and PHP 1b. The demonstration of UPD also explained the broad range of methylation aberration encompassing all four DMRs in the GNAS imprinting cluster.

The proband showed MLID including *DIRAS3* and *PLAGL1* along with IC2 and *GNAS* cluster which are loci relevant to his clinical features. The underlying mechanisms associated with MLID are not elucidated yet, though secondary epimutations resulting from pathogenic variants in certain genes such as *ZFP57, NLRP2* and *PADI6* have been identified to be involved in a few patients with transient neonatal diabetes mellitus (TNDM) and BWS, respectively [20,21,23]. However, no pathogenic variant has been identified in *ZFP57* implicated in BWS and PHP1b patients with MLID to date [14,34,35], and pathogenic variants in *NLRP2* and *PADI6* were reported as a potential cause of MLID in only few familial BWS cases without corroboration by other studies yet [21]. Alternatively, it is possible that in our patient the maternal UPD 20 has led to a similar recessive disorder for a variant that is heterozygous in the mother as it is known that some consequences of isodisomy include unmasking of recessive diseases [36]. That is, it is theoretically possible that the UPD20 is relevant to the MLID in our case.

For the clinical diagnosis of PHP1b, elevated PTH and/or hypocalcemia need to be documented by laboratory evaluation. However, PTH levels gradually increase as patients grow older and PTH resistance appears to develop beyond 1–2 years old in PHP1b at least [37,38]. Therefore, the mean age of diagnosis for PHP1b is after 10 years of age even in patients presenting with clinical symptoms, [39]. This differs from BWS which is often diagnosed based on clinical characteristics present in neonatal and infant periods. For these reasons, PHP1b may have been overlooked in a certain fraction of patients with MLID. All three reported patients with BWS and PHP1b including our patient were diagnosed at similar chronological ages; that is BWS was diagnosed in infancy, but PHP1b was diagnosed over the age of 12 years [19,22] None of three patients experienced hypocalcemic tetany or seizures.

With respect to the growth pattern in our patient, the influence of both BWS and PHP on growth velocity is likely complex. AHO has been thought to be a typical and confined character for PHP1a. AHO is a clinical entity which encompasses heterogeneous clinical findings such as short stature, obesity, round face, brachydactyly, subcutaneous calcification, and variable degrees of mental retardation [3,4]. However, AHO features vary from mild to severe, and several PHP1b patients with *GNAS* methylation alterations also have been reported to have AHO features [34,40,41,42]. These reports suggest that there is clinical and molecular overlap among PHP subtypes [3]. Unlike the other two patients with both BWS and PHP1b, our patient had mild learning disability and stunted height velocity in his early teens. Intellectual disability with variable degrees and short stature are included among the main clinical characteristics of AHO. Our patient could represent another case of PHP1b with mild AHO features, even if skeletal structural abnormality or subcutaneous calcification was not observed. There have also been some reports suggesting that developmental delay may occur more frequently in BWS patients with MLID than in those without MLID [14,43]. Thus, MLID may also be a contributing factor to the mild learning disability shown in our patient.

In terms of BWS, most BWS children show rapid growth in early childhood, however height velocity appears to slow around age 7–8 years and bone age is commonly advanced. Adult height typically remains at the upper range of normal [2]. However, our patient showed delayed pubertal development by about two years compared to his peers rather than early puberty, and no evidence of advanced bone age was noted. Constitutional delay in growth and puberty might also contribute to his stunted growth shown in his early teens.

## 5. Conclusions

In summary, BWS and PHP1b were diagnosed in our male patient at chronological ages typical for each of these disorders. Extensive molecular analyses identified patUPD20 and IC2-LOM as the causes of the patient’s PHP1b and BWS diagnoses, respectively as well as MLID. These studies underscore the importance of evaluating parental genomic contributions in cases of ID and putative MLID using advanced molecular genetic techniques to determine the underlying mechanisms relevant to the occurrence of two or more IDs in the same patient.

## Figures and Tables

**Table 1:** Results from detailed analysis of CpG sites overlapping known imprinted loci using 450K methylation array.

Gene Symbol	Chromosome Location	Number of CpG Sites	Controls (*n* = 6)		Patient
			Average	2SD	
*DIRAS3-1*	1p31.3	27	0.56	0.04	**0.26**
*PLAGL1*	6q24	15	0.57	0.06	**0.18**
*KCNQ1OT1*	11p15.5	28	0.56	0.05	**0.19**
*NESP55*	20q13.32	22	0.55	0.06	**0.92**
*GNASAS*	20q13.32	59	0.52	0.07	**0.10**
*GNASXL*	20q13.32	6	0.56	0.04	**0.05**
*GNAS1A*	20q13.12	40	0.57	0.06	**0.12**

Methylation values beyond average ± 2SD of controls are represented in bold.

**Table 2:** Summary of the reported patients with coexistence of BWS and PHP1b.

	Case 1	Case 2	Proband
Sex	F	F	M
Age (year) at diagnosis of BWS	0.5	at birth	0.3
Age (year) at diagnosis of PHP1b	16	12.4	14
Phenotypes for BWS	macrosomia, umbilical hernia	macroglossia, macrosomia, umbilical hernia, hypoglycemia, hemihypertrophy	macroglossia, macrosomia, umbilical hernia, ear crease, hypoglycemia
Phenotypes for PHP1b	hypocalcemia, hyperphosphatemia, parathyroid hormone resistance, no AHO	hypocalcemia, hyperphosphatemia, parathyroid hormone resistance, no AHO	hypocalcemia, hyperphosphatemia, parathyroid hormone resistance, stunted growth, mild learning difficulty
Phenotype-related DMRs	BWS: LOM-IC2 PHP1b: LOM-*AS, XL, A/B*, GOM-*NESP*	BWS: LOM-IC2 PHP1b: LOM-*AS, XL, A/B,* GOM-*NESP* (patUPD20 excluded)	BWS: LOM-IC2 PHP1b: patUPD20 (LOM-*AS, XL, A/B,* GOM-*NESP*)
Other associated DMRs with MLID	not examined	*DIRAS3, FAM50B, PEG1/MEST, RB1*	*DIRAS3, PLAGL1*
Conception	natural	natural	natural
Maternal /paternal age at birth (years)	unknown	33/34	39/39
Mutation of a gene for MLID	not examined	not identified in *ZFP57, NLRP2, NLRP7, KHLC3L, NLRP5*	not examined
Reference	[22]	[19]	this report

## Data Availability

The data presented in this study are available on request from the corresponding author. The data are not publicly available due to institutional restrictions.

## Supplementary Materials

The following are available online at https://www.mdpi.com/2073-4425/12/2/172/s1, Table S1: DNA methylation levels at imprinted loci.
